# Tracking the geographical origin of *Plasmodium falciparum* causing a rare severe case of malaria imported into Palestine, a zero-indigenous case area

**DOI:** 10.1186/s12879-023-08583-4

**Published:** 2023-09-18

**Authors:** Anas Al-Jawabreh, Suheir Ereqat, Amer Al-Jawabreh, Ahmed Al-Jawabreh, Hanan Al-Jawabreh, Abedelmajeed Nasereddin

**Affiliations:** 1 Faculty of Medicine, Al-Quds University, Abu Deis, East Jerusalem, Palestine; 2 Biochemistry and Molecular Biology Department, Faculty of Medicine, Al-Quds University, Abu Deis, East Jerusalem, Palestine; 3 Arab American University, Jenin, Palestine; 4 Leishmaniases Research Unit, Jericho, Palestine; 5 Palestine Medical Complex, Ministry of Health, Ramallah, Palestine; 6 Al-Quds Bard College, Al-Quds University, Abu Deis East Jerusalem, Palestine

**Keywords:** Malaria, *Plasmodium falciparum*, Palestine, Jericho, Next-generation sequencing, Ring form, Whole genome sequence, Phylogenetic tree, *msp-1*

## Abstract

**Background:**

Malaria cases in non-endemic zero-indigenous case areas are most likely to have been imported whatever of the route of importation. In countries recently declared malaria-free and now without local transmission, imported cases remain a threat to re-introduction of the disease and a burden on the health system.

**Case presentation:**

Three days after returning from a long trip to malaria- endemic countries; Abyei-Sudan, Chad and Uganda, a 41-year-old male resident from Jericho, Palestine, suffered paroxysms of fever, general fatigue, myalgia, arthralgia, headache, and a strong desire to vomit. Thin and thick Giemsa-stained blood smears were prepared and examined microscopically using oil immersion. Immature trophozoites (ring forms) were seen to parasitize approximately 10% of the erythrocytes revealing hyperparasitemia equivalent to > 100,000 parasites/ µl indicating severe malaria [1, 2]. The double chromatin configuration (headphones) and accolé (applique) position are both indicative of *Plasmodium falciparum* infection. The 18S rRNA- PCR targeting the rPLU6-rPLU5 region was used to confirm the diagnosis. The next-generation sequencing (NGS) method was carried out according to the manufacturer’s instructions (Illumina® DNA Prep, (M) Tagmentation kit (20060060), Illumina) to identify *Plasmodium* spp. Furthermore, NGS produced a whole-genome sequence of 22.8Mbp of the 14 chromosomes and 25Kbp of the apicoplast. A BLAST search of the apicoplast DNA and selected chromosomal DNA revealed that *P. falciparum* was the causative agent. The merozoite surface protein-1 (*msp-1*) was used to construct a phylogenetic tree of 26 *P. falciparum*, including the one isolated from the patient from Jericho, which clustered with the Sudanese isolate indicating genetic relatedness between the two.

**Conclusion:**

The travel history together with signs and symptoms of malaria, followed by prompt diagnosis using conventional microscopic inspection of Giemsa-stained films together with molecular DNA tracking tools like *msp-1* were key means in tracking the place of origin of infection in the case of travel to multiple destination.

## Background

Malaria is a potentially lethal infection caused by an apicomplexan parasite of the genus *Plasmodium.* It is a vector-born disease transmitted by female mosquitoes of the genus *Anopheles*. Of the four clinicallysignificant species in the genus, *Plasmodium falciparum* is the most widely distributed in Africa and having the most severe clinical consequences [3, 4]. The global burden of malaria, morbidity and mortality, is considered to be the highest among all the parasitic diseases with 247 million cases and 619,000 deaths per annum [4]. Sub-Saharan Africa is the geographical region affected most bearing 95% of the deaths with 50% of them occurring in the following four African countries, Nigeria, The Democratic Republic of Congo, Uganda, and Mozambique [4].With exception of Saudi Arabia and Yemen, all the Middle Eastern countries are free of endogenous cases of malaria, however, with most of these countries reporting imported cases [5]. At the turn of the 20th century, Palestine was described as one of the most highly malarious countries in the world [6]. Reports as early as 1880 confirmed that malaria caused by *P. falciparum* and *P. vivax* was a major public health problem. The malaria infection rate ranged from 50, 20, 7, and 0.7% in the years 1905, 1913, 1922, and 1944, respectively [7–9]. Six to eight species of anopheline mosquitoes were collected as larvae and adults from Palestinian marshes, swamps, and springs [6, 10]. Several attempts were conducted to eliminate malaria, starting during Ottoman rule, then during British and Jordanian rule that led to the eradication of the disease by end of the 1960 [6, 8, 10, 11]. According to International Classification of Diseases for Morbidity and Mortality Statistics, 11th Revision, v2022-02 (ICD11) a disease caused by *P. falciparum* is characterized by fever, chills, headache, myalgia, arthralgia, weakness, nausea, anemia, jaundice, vomiting, or diarrhea. Other more severe manifestations include splenomegaly, hypoglycemia, pulmonary or renal dysfunction, and neurological changes such as mental confusion and seizures followed by coma and death. Here the geographical origin of a rare case of malaria imported into Palestine was tracked using next-generation sequencing and population genetics.

## Case presentation

**Fig. 1:**
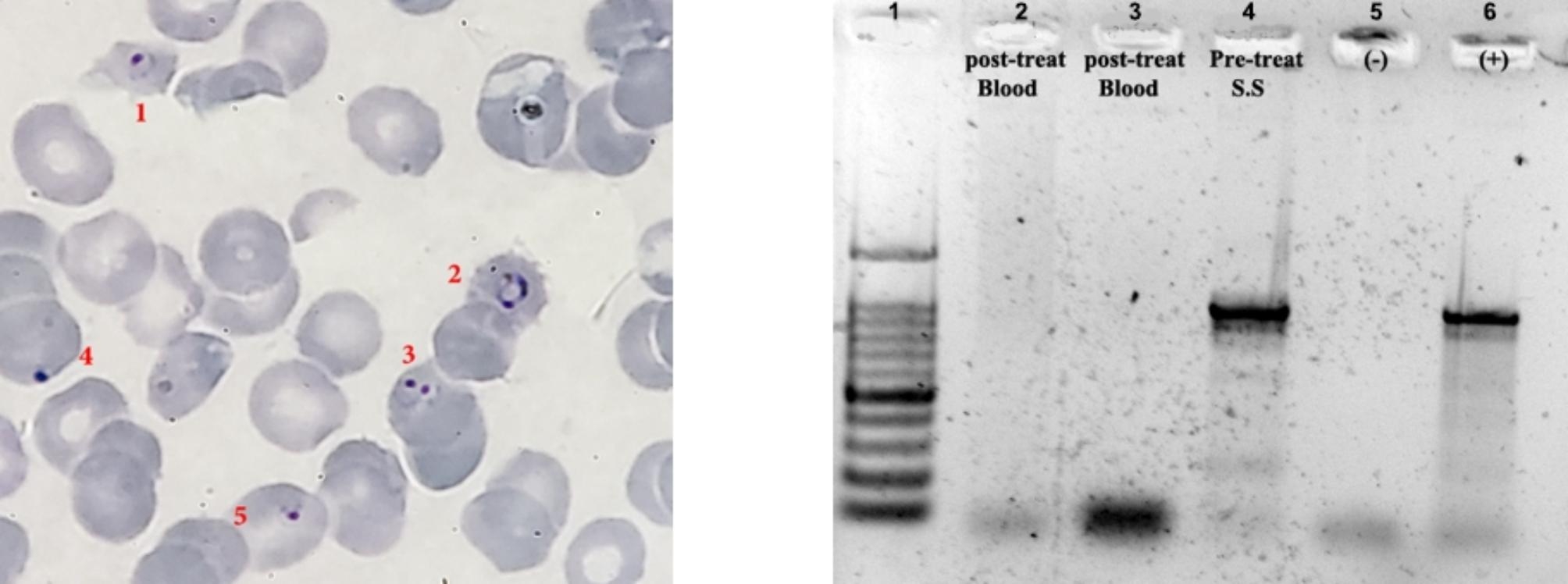
(**a**) A thin blood film made from blood from the case of malaria and stained with Giemsa’s stain as seen microscopically at X1000 magnification. Red numbers indicate erythrocytes infected with immature trophozoites (ring form) of *Plasmodium* spp. Erythrocyte 3 shows the double chromatin configuration (headphones) and erythrocyte 4 is in the accolé (applique) position, both of which are indicative of an infection of *Plasmodium* spp.(**b**) An agarose gel showing the amplification of region rPLU6-rPLU5, the region of the 18S rRNA gene, showing: 1, the 100 bp marker; 2 and 3, two samples, one after one week and the other after two weeks post-treatment; 4, pre-treatment sample during the acute phase; 5 and 6, negative and positive controls, respectively

A 41-year- old Palestinian man from Jericho worked as an engineer with an international institution in Abyei, a disputed area between Sudan and South Sudan. In 2018, he moved from Abyei to Eastern Chad and, spent a few days on an official mission. Then, he flew to Kampala, the capital of Uganda, where he spent one week at the institution headquarters and finally, flew home to Palestine through Dubai and Amman, Jordan. Three days after reaching his final destination in Jericho, Palestine, he started to suffer a high fever with chills and sweating. These paroxysms of fever, chills and rigor were accompanied by general fatigue, a headache and strong need to vomit despite not having eaten since the start of the symptoms. In addition, he suffered myalgia and arthralgia. He was admitted to the Jericho Government Hospital for a clinical follow up where his clinical history revealed incompliance with the recommended prophylactic treatment when using Malarone® (GlaksoSmithKine, GSK). Urinalysis, stool analysis, complete blood count, and examination of blood films were done, all of which were normal. Thick and thin blood films were prepared and examined in between the paroxysms. Blood films were stained for one hour with Giemsa’s stain made with 1:40 phosphate buffered water according to Garcia [12]. Blood films revealed the presence of the parasitaemia seen as immature trophozoites (ring forms) and indicating malaria (Fig. 1a). According to World Health Organization (WHO) malaria grading system, the case was considered hyperparasaetimic owing to the presence of >10 infected RBCs per 100 normal RBCs in an oil immersion field equivalent to > 100,000 parasites/µl indicating severe malaria (Fig. 1a). In some maturing trophozoites ring forms with thicker cytoplasm were seen occasionally in stained thin smears. DNA was extracted from the stained smears, using a QIAamp® DNA Mini Kit according to manufacturer’s instructions (Qiagen, GmbH, Hilden, Germany). Genomic DNA was used for PCR amplification, according to Snounnou et al., which amplified the 18S rRNA gene [13]. Briefly, in 25-µl-reaction; was conducted, 0.25µM of each of the rPLU6/rPLU5 primer pair, in a Syntezza Bioscience reaction tube (Jerusalem). A known *Plasmodium falciparum* DNA (Provided by Prof. Ron Dzikowski), 0.04ng/µl (1ng/25µl reaction) of DNA and PCR grade water was used as negative control. A PCR product of approximately 1200 bp was obtained from the DNA of the infecting parasites, which was equivalent to the positive control after running the samples on a 2% agarose gel and staining with ethidium bromide, using the Biorad gel doc XR documentation system (Bio-Rad Laboratories Inx, USA) (Fig. 1b). In addition to confirm the positive results of the blood films and determine the PCR was meant to assess suitability, the quantity and quality of the DNA for the WGS.

**Fig. 2:**
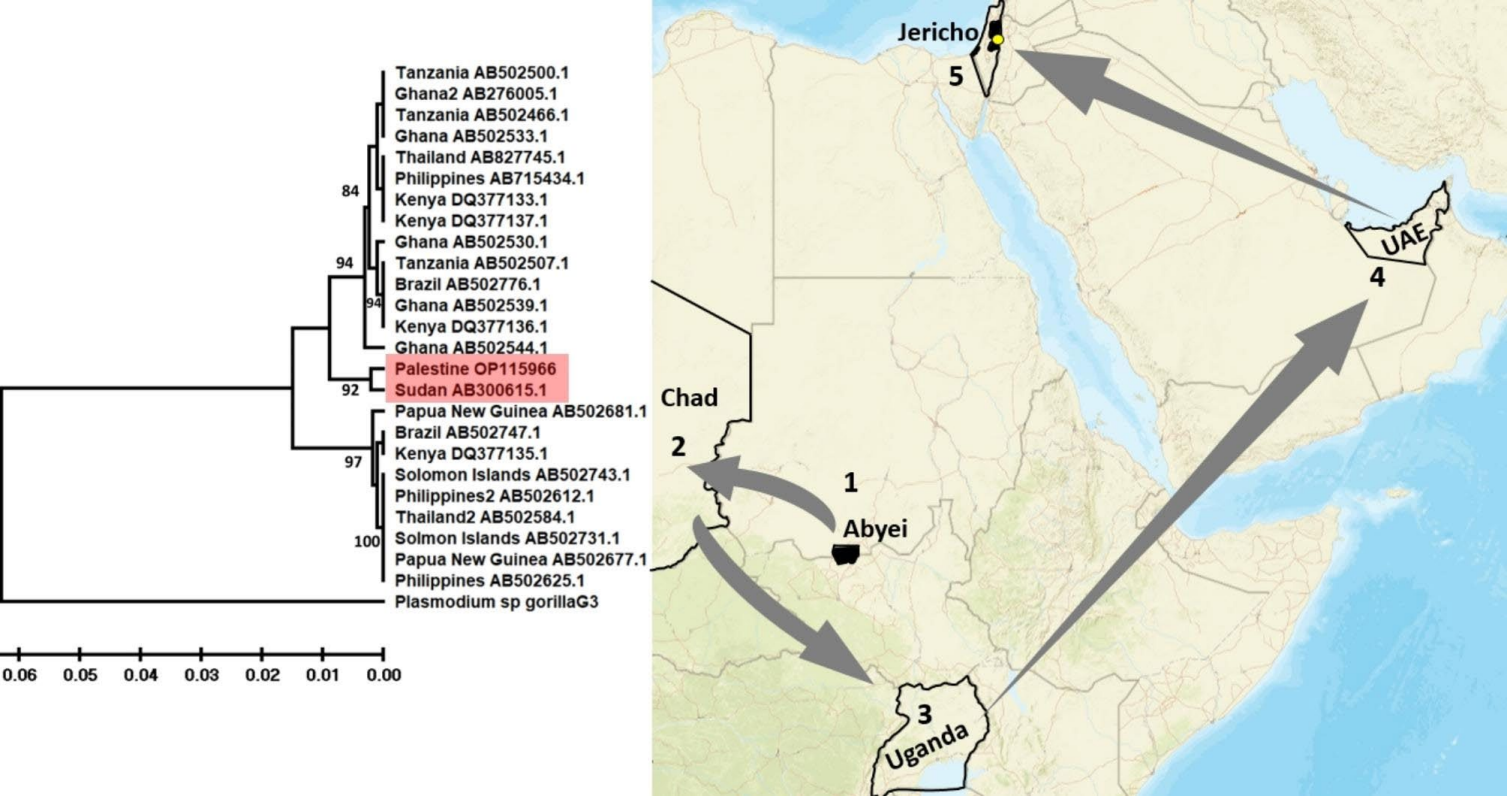
UPGMA phylogenetic tree was constructed using MEGA-X free software, and showing relationships between the sequence of the *msp-1* DNA of the isolate of the *P. falciparum* imported into Jericho and the 25 *msp-1* DNA sequences from other strains of *Plasmodium* retrieved from the GenBank. *Plasmodium* spp. *msp-1* DNA sequence isolated from gorilla clade G3 (Accession number LT969558.1) was used as an out-group. A bootstrap value of 1000 replicates was used to ensure robustness of the tree and shown as percentages at tree nodes. The tree was drawn to scale with branch lengths given in the same units as those of the evolutionary distances used to infer the phylogenetic tree [18–21]. The map shows the patient’s travel through five different geographical regions, two to three weeks before the appearance of the symptoms suggestive of malaria. Map was generated with Epi Info™ CDC-free software

The WGS analysis exposed 14 chromosome (22.8Mbp) and an apicoplast (25Kbp) DNA sequences [14]. The whole genome sequence (Accession numbers CP101623-36) and apicoplast sequence (Accession number OP661153) were deposited in the GenBank. Using a BLAST search, the apicoplast DNA,*msp*-1 (Accession number OP115966), *msp-2* (Accession number OR198912), and any selected chromosomal DNA showed that *P. falciparum* caused the imported case of malaria. Based on the WHO recommendations and other studies, population genetics of the genus *Plasmodium* was based on the hypervariable region (556bp) of the merozoite surface protein I (*msp-1*) owing to its extensive polymorphism, stability throughout the life cycle, the availability of the widely used data and the easy distinguishable [15–17]. The Jericho patient *msp-1* sequence was retrieved from the WGS resulting from this report. The phylogenetic tree based on the *msp-1* of 26 isolates of *P. falciparum* from different countries was constructed. This include one from the resident of Jericho. The tree showed that this isolate clustered with the Sudanese isolate (Fig. 2). In accordance with the solid laboratory diagnosis of malaria caused by *P. falciparum*, the patient was treated for three days with tablets (artemether-lumefantrine) of the antimalarial drug Coartem® (Novartis) as the drug of choice for acute malaria. The patient ingested four tablets every eight hours on day one, and four tablets every 12 h on days two and three. His condition improved with immediate resolution of all the acute symptoms, which was confirmed on examining thin blood films and employing PCR testing (Fig. 1b) one week and two weeks post-treatment.

## Discussion

This quite severe case of malaria was diagnosed in Jericho, Palestine, a zero-indigenous case area and declared by the WHO as a country free of malaria transmission [22], which made it difficult to decide the exact etiology based solely on intermittent high fever. Other diseases in the area causing fever, e. g. visceral leishmaniases, meningitis, pneumonia, malignant tumors, and viral hepatitis, were possible suspects. The patient’s travel history to and in African, malaria-endemic regions, followed by episodes of fever made malaria highly suspect and examining stained blood films followed by employing PCR and NGS methods confirmed malaria caused by *P. falciparum*, the most severe form of malaria. This proved the efficacy of using NGS and *msp-1* DNA in tracking the geographical origin of the parasites seen in the stained smears. The main limitation was the need of a high depth of sequencing coverage (6.2x) to cover the WGS of *P. falciparum* (22.8Mbp), which is within a much larger amount of human host DNA (3.2Gbp). Regarding this case, local transmission was ruled out owing to the lengthy incubation period of malaria caused by *P. falciparum* of at least two weeks. Also, in theory, local transmission could not have occurred, owing to the absence of the main vectors of *P. falciparum*, *i. e., Anopheles gambiae* and *Anopheles funestus*, as evidenced by the complete absence of anopheline species during insect trapping campaigns in Palestine in the last two decades [23–25]. A possibility of transmission is through airport malaria, in which a traveler hopped between several airports, including ones in countries where malaria is endemic (Fig. 2). There have also been reports of ‘baggage malaria’ where anopheline mosquitoes find their way into and surviving in luggage and goods being transported from African countries where malaria is endemic to countries devoid of malaria and transmitting malaria there [26–30]. The other possibility, as is the situation described in this article, the patient is an extensive traveler [31]. In this case, the clinical history ruled out the further routes of transmission, e. g., such as blood transfusion, nosocomial (hospital-acquired) infection, post-transplant, parenteral such as drug abuse needle sharing, being immuno-compromised, and recrudescence (relapsing) after having had malaria [32, 33]. In theory, the patient could have contracted his infection in one of the three African countries he visited Sudan, Chad, and Uganda. Also, airports in these countries and those of Dubai and Amman may have been the venue of his infection. Population genetics was used to track the origin of his infection by correlating the DNA of *P. falciparum* extracted from his infected blood with DNA from reference strains from other countries around the world. The phylogenetic tree based on the *msp-1* of all the samples used clustered his causative agent with a Sudanese one, indicating, most likely, that the patient contracted his infection in the Abyei region of Sudan and not from any other region he travelled to. Imported cases of malaria from regions of malaria endemicity to those of non-endemicity are common. From 1990 and early 2021, 35 cases of malaria were recorded in Palestine but without mentioning whether the diagnosis was only symptom-based or also laboratory-confirmed, or if these cases were imported or autochthonous [34]. In 2005, the Palestinian Ministry of Health declared that it has completely controlled many infections including malaria [34]. Ten years later, the WHO declared Palestine a country free of local transmission of malaria [22]. The case imported into Jericho and described here and whole-genome sequenced, permitting species identification and tracking of the cause and origin of the infection, is the only one dealt in this way in Palestine so far.

## Conclusion

Cases of malaria imported into non-endemic countries, Palestine, is a challenge to their health system’s preparedness to diagnose and treat such cases. The careful taking of clinical histories, laboratory confirmation of cases including the identification of the infectious agents, using microscopy and molecular-based methods, and tracking the origin of the infection are recommended to enable timely life-saving treatment and to understand the disease epidemiology. Travelers to and from endemic countries should be dealt with according to WHO international health regulations and their amendments [35]. Monitoring for the presence of species of *Anopheles* in recently declared malaria free areas is crucial in preventing re-introduction of the disease.

## Data Availability

The datasets used and/or analyzed during the current study are available from the corresponding author on reasonable request.

## Abbreviations

PCR: Polymerase chain reaction
NGS: Next-generation sequencing
WGS: Whole-genome sequence
*msp-1*: Merozoite surface protein-1
UPGMA: Unweighted Pair Group Method with Arithmetic Mean
RBC: Red blood cell
